# Cross-trait genetic architecture between breast cancer and psychiatric disorders

**DOI:** 10.1016/j.isci.2026.116767

**Published:** 2026-07-13

**Authors:** Canzhou Wang, Jingxi Hu, Yan Lei, Jing Li, Yaochen Zhang, Kai Hu, Liu Liu, Xin Su, Xinxu Wang, Jinqi Wang, Yanhong Wang, Hongyan Jia

**Affiliations:** 1 Department of First Clinical Medicine, Shanxi Medical University, Taiyuan, China; 2 Department of Breast Surgery, First Hospital of Shanxi Medical University, Taiyuan, China; 3 Department of Second Clinical Medicine, Shanxi Medical University, Taiyuan, China; 4 School of Basic Medical Sciences, Shanxi Medical University, Taiyuan, China

**Keywords:** breast cancer, psychiatric disorders, GWAS, genetic overlap, pleiotropic genes

## Abstract

Breast cancer (BC) and psychiatric disorders are epidemiologically linked, raising the possibility of shared genetic influences. Based on the summary statistics from the genome-wide association studies (GWAS), the genetic correlation and overlap between psychiatric disorders and BC were investigated. Shared pleiotropic loci and genes were identified via cross-trait analyses. Functional annotations and tissue-specific enrichment were carried out to determine potential associations. A total of 7,274 pleiotropic single nucleotide polymorphisms (SNPs) were identified by cross-trait analyses. Furthermore, 156 shared genomic risk loci were identified by annotation, of which 19 passed the colocalization test. The gene-level analysis discovered 142 pleiotropic genes, among which *MRTFA* and *FGFR2* were identified in most trait pairs. Pathway enrichment highlighted positive regulation of RNA. Finally, protein quantitative trait locus (pQTL)-based summary-data-based Mendelian randomization (SMR) analyses further prioritized plasma proteins associated with both phenotypes. These findings delineate a common genetic basis for BC and psychiatric conditions, offering insights into their comorbidity.

## Highlights

• Two BC subtypes and eight psychiatric disorders share genetic architecture
• 7,274 pleiotropic SNPs and 19 high-confidence loci were identified
• MRTFA, FGFR2, DNPH1, FURIN, and GATAD2A were prioritized
• ERPBC-specific RNA metabolism enrichment suggests regulatory convergence

## Graphical abstract

**Figure:**
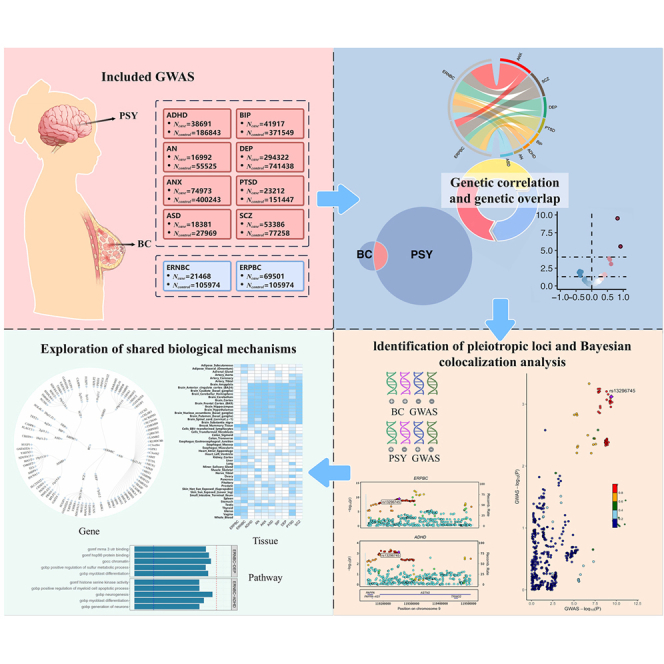

## Teaser

Human genetics; Precision medicine; Genetics; Genomics; Bioinformatics; Computational bioinformatics; Systems biology

## Introduction

Breast cancer (BC) is a heterogeneous malignancy classified into estrogen receptor (ER)-positive BC (ERPBC) and ER-negative BC (ERNBC) subtypes. It has been estimated that each year, there are approximately 11.6% of new cancer cases and 6.9% cancer-related deaths worldwide.^1^ ERPBC is strongly influenced by hormonal signaling and differs from the ERNBC, which is often characterized by aggressive biology and limited therapeutic options.^2^ The progression of BC is significantly influenced by certain risk factors, such as obesity, hormonal regulation, and mental disorders.^3^^,^^4^^,^^5^ Recently, the association between psychiatric disorders and BC has garnered increasing attention. Psychiatric disorders, including attention deficit hyperactivity disorder (ADHD), anorexia nervosa (AN), anxiety disorder (ANX), autism spectrum disorder (ASD), bipolar disorder (BIP), depression (DEP), post-traumatic stress disorder (PTSD), and schizophrenia (SCZ), have overlapping symptomatology and shared genetic vulnerabilities. Epidemiological studies have indicated bidirectional comorbidities between cancer and psychiatric disorders, potentially mediated by dysregulated immune responses, neuroendocrine pathways, or oxidative cell cycle regulation.^6^^,^^7^^,^^8^^,^^9^^,^^10^^,^^11^ Although the effects of lifestyle factors or treatment often confound the epidemiological associations, the shared genetic architecture between psychiatric disorders and BC subtypes warrants a comprehensive investigation to identify the underlying mechanisms regulating this comorbidity.^12^

Genome-wide association studies (GWAS) have identified hundreds of risk loci for BC subtypes and psychiatric disorder traits. For instance, Lu discovered 19p13 (*GATAD2A*) as a shared locus associated with BC and SCZ.^13^ Furthermore, DEP and BC were found to share common biological mechanisms at the gene and tissue levels, such as inflammatory and immune responses.^8^ A Mendelian randomization analysis revealed no evidence of a causal relationship between five psychiatric disorders and BC. This finding indicates that the relationship between psychiatric disorders and BC requires explanation through mechanisms beyond vertical pleiotropy.^14^ However, several research gaps remain; a multi-level priority screening framework has yet to be established, the extent of genetic overlap between ER-defined BC and psychiatric disorders is unknown, the shared genetic loci and biological pathways remain unidentified, and the functional relevance of pleiotropic variants is uncharacterized.

Recent advances in statistical genetics have provided multiple methods for comprehensive genome-wide cross-trait analyses. These methods can fill the aforementioned gaps, identify the etiological associations, and provide novel insights into the underlying biological mechanisms. Therefore, this study employed a causal mixture model (MiXeR) to quantify genome-wide polygenic overlap and determine shared causal variants with mixed effect directions. Furthermore, the pleiotropic analysis cross-trait omnibus (PLACO) test framework and latent heritable confounder Mendelian randomization (LHC-MR) were employed to investigate horizontal pleiotropy (i.e., genetic variation affects two phenotypes) and vertical pleiotropy (i.e., genetic variation affects one phenotype and causes another phenotype to occur), respectively. Moreover, gene and pathway enrichment analyses were performed via multi-marker analysis of GenoMic annotation (MAGMA) to assess the biological mechanisms underlying shared associations. Using protein quantitative trait locus (pQTL) data, we performed summary-data-based Mendelian randomization (SMR) analysis to genetically prioritize plasma proteins related to BC and psychiatric disorder phenotypes.

This genome-wide pleiotropic association study performed multi-level pairwise trait analyses using GWAS summary data of two BC subtypes and eight psychiatric disorders. This study aims to (1) quantify genome-wide genetic correlations, local genetic correlations, and genetic overlaps beyond mere correlations; (2) evaluate pleiotropic loci and genes; and (3) elucidate biological pathways and tissue-specific mechanisms. The acquired findings will enhance our understanding of the etiology of these comorbid conditions and provide a theoretical basis for the development of targeted prevention strategies for high-risk populations with both psychiatric and oncological diagnoses.

## Results

**Figure 1:**
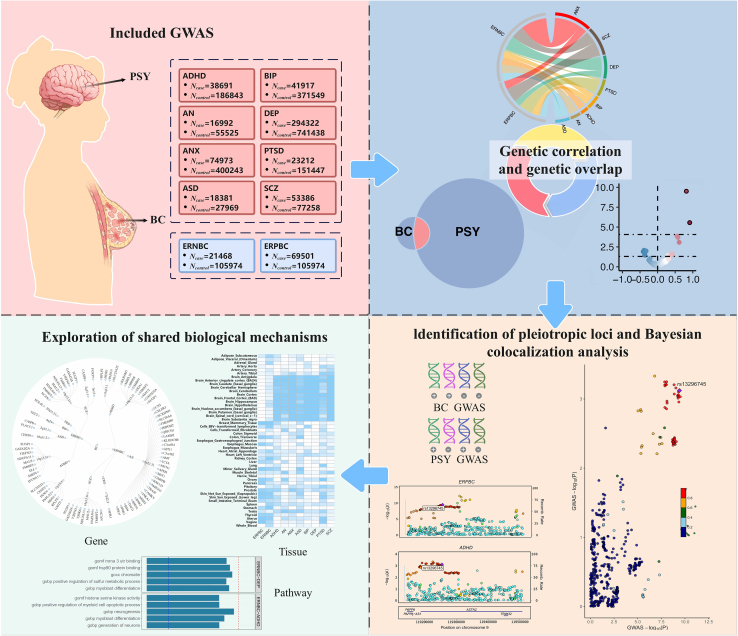
Study overview The workflow summarizes the major analytical steps of this study, including genome-wide association studies (GWAS) summary statistics collection, quality control, genome-wide and local genetic correlation analyses, MiXeR-based genetic overlap analysis, pleiotropic analysis cross-trait omnibus (PLACO)-based identification, functional annotation, and colocalization, tissue-specific enrichment, gene-level and pathway-level analyses. BC, breast cancer; PSY, psychiatric disorders; ERPBC, estrogen receptor-positive breast cancer; ERNBC, estrogen receptor-negative breast cancer.

A study overview is presented in Figure 1. In this study, high-confidence pleiotropic loci were defined as PLACO-based FUMA genomic risk loci that additionally showed colocalization evidence between paired traits (PP.H4 > 0.7), while high-priority pleiotropic genes were defined as genes supported by convergence across complementary gene-level analyses and previous experimental evidence.

### Genome-wide genetic correlation analysis

Based on the GWAS summary statistics, linkage disequilibrium score (LDSC) regression was employed to identify a genome-wide genetic correlation overview. Furthermore, univariate analysis was carried out to evaluate the h^2^SNP of BC and psychiatric disorders, and the results revealed that the eight psychiatric disorders exhibited relatively higher heritability, ranging from 0.020 to 0.353, with SCZ having the highest heritability (standard error [SE] = 0.013) and PTSD the lowest (SE = 0.004). Among the two BC subtypes, ERPBC (h^2^SNP = 0.130, SE = 0.012) indicated heritability double that of ERNBC (h^2^SNP = 0.070, SE = 0.007).

The bivariate analysis was carried out to assess the genome-wide genetic correlations (rg) between BC and psychiatric disorders. It was observed that 7 of 16 trait pairs had nominally significant genetic correlations (*p* < 0.05), among which all of them exhibited a positive correlation (rg ranging from 0.050 to 0.199). Furthermore, of the 16 trait pairs, 4 pairs [ANX-ERNBC (*p* = 4.19E−05), DEP-ERNBC (*p* = 7.00E−04), SCZ-ERNBC (*p* = 2.90E−03), and SCZ-ERPBC (*p* = 4.11E−06)] were significantly genetically correlated after Bonferroni correction (Table S2).

### Genetic overlap in MiXeR

**Figure 2:**
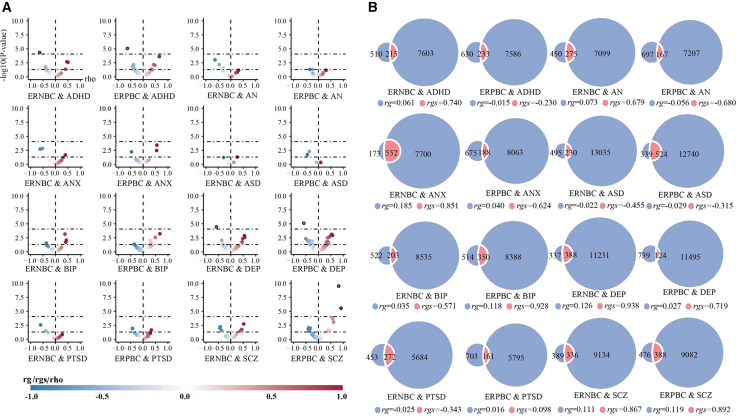
Local genetic correlation and genetic overlap (A) Volcano plots of local analysis of [co] variant annotation (LAVA) local genetic correlation coefficients (rho, *y* axis) against −log10 (*p*-values) for each pairwise analysis. (B) Venn diagrams showing MiXeR results of the estimated number of variants shared between BC and psychiatric disorders. The size of the circles reflects the degree of polygenicity.

Genetic correlation can identify genetic relationships between phenotypes; however, it cannot assess whether near-zero correlations reflect (1) offsetting variant effects (concordant and discordant) or (2) true genetic independence. To resolve this ambiguity, integrative analytical frameworks with complementary assumptions are required to estimate genetic overlap (Figures 2 and S1).

Univariate MiXeR quantified the heritability, polygenicity, and discoverability for BC and psychiatric disorders. It is revealed that all the phenotypes had good model fit except PTSD, as indicated by positive AIC values. Furthermore, BC had lower polygenicity (ERNBC [*n* = 725] and ERPBC [*n* = 864]), higher discoverability (ERNBC [2.21E−04] and ERPBC [2.05E−04]), and relatively low SNP heritability (ERNBC [0.103] and ERPBC [0.114]). Among the eight psychiatric disorders, SCZ had the highest heritability (h2 = 0.380), while PTSD had the lowest heritability (h2 = 0.030), consistent with LDSC results (Table S3).

The bivariate MiXeR analysis showed multiple shared variants in each pair. All trait pairs showed good to medium model fit with positive AICbest vs. max. For ERNBC-related trait pairs, the largest genetic overlap was observed between ERNBC and ANX (Dice = 0.123, SE = 0.027). There is medium genetic overlap between ERPBC and ADHD (Dice = 0.054, SE = 0.036) among ERPBC-related trait pairs. The proportion of concordant effect among these variants was 0.417, which aligns with the weak genetic correlation (rg = −0.015, rgs = −0.230). The comparison between ERNBC and ERPBC indicated similar levels of genetic overlap with most psychiatric traits. The shared genetic architecture between ERPBC and ADHD greatly influenced mixed factors, which can explain the weak genetic correlation (rg = −0.001, SE = 0.028) observed in LDSC. In general, MiXeR results indicate widespread genetic overlap between BC and psychiatric disorders; however, this only reflects statistical co-architecture of causal variants, and further research is needed to determine direct functional convergence at the molecular or cellular level.

### Estimating local genetic correlations

Genome-wide genetic correlation may not be represented due to the equal number of positive and negative effects within their regions. Therefore, to identify local genetic correlation, LAVA was performed to estimate local genetic correlation (Figure 2). The findings identified the local heritability of each genetic region, which supported the previous estimations of heritability. In total, 24 loci were identified as significant (false discovery rate [FDR] <0.05), with 33.3% showing negative and 66.7% showing positive loc-rgs. There were no shared genetic loci between ASD and BC, AN, and ERPBC, which might be because LAVA only identifies loci with very strong associations compared with MiXeR, which highlights loci that are more likely to be statistically significant (Tables S4 and S5).

### Causal analyses

The above results indicated a shared genetic basis between psychiatric disorders and BC. LCV was performed to identify the potential causal associations (vertical pleiotropy) of observed genetic correlations. LCV did not support significant evidence for partial genetic causality across the 16 trait pairs. Furthermore, the LHC-MR approach was employed to assess the reproducibility of partial causal associations between trait pairs. The LHC-MR analysis suggested three significant genetically inferred directional associations: ANX-ERNBC (odds ratio [OR]: 1.508; 95% confidence interval [CI]: 1.245–1.826), PTSD-ERNBC (OR: 1.488; 95% CI: 1.264–1.752), and ERPBC-PTSD (OR: 1.105; 95% CI: 1.045–1.168). No evidence of bidirectional genetically inferred directional associations was observed. Conventional MR analyses were also performed in the LHC-MR framework. MR results showed that of these three trait pairs, only ERPBC-PTSD showed statistically significant results in the inverse variance weighted method with no strong bias. Overall, causal analyses showed that vertical pleiotropy is weak between BC and psychiatric disorders (Table S6; Figure S2).

### Identifying pleiotropic loci through cross-trait analysis

After identifying the genetic correlation and overlap between BC and psychiatric disorders, PLACO was employed to investigate potential pleiotropic SNPs influencing both diseases (i.e., horizontal pleiotropy). To avoid overinterpretation of all PLACO-significant variants, we prioritized cross-trait signals using a prioritization framework, including PLACO-significant SNPs, FUMA genomic risk loci, and high-confidence pleiotropic loci supported by colocalization evidence. A total of 7,274 significant SNPs were identified at the threshold of *p* < 5.00E−08. These 7,274 SNPs were submitted to FUMA for comprehensive annotation (Tables S7, S8, S9, and S10). The results identified 156 genomic risk loci spanning 68 unique chromosomal regions. Furthermore, 126 pleiotropic loci, including 19p13.11, indicated genetic signals for multiple trait pairs. Bayesian colocalization analyses revealed that 19 of the 156 potential pleiotropic loci had PP.H4 > 0.7 (Table 1) and were therefore prioritized as high-confidence pleiotropic loci. Among these, 13 loci were also supported by gene-level analyses (Table S11), representing further prioritized pleiotropic candidate loci. The 19p13.11 locus was identified as pleiotropic for all ADHD- and SCZ-associated trait pairs, and these traits had significant colocalization (PP.H4 ranging from 0.731 to 0.970). Among all these pleiotropic loci, 80 (51.3%) had inconsistent effects, which partly explains MiXeR analysis results. ANNOVAR category annotation revealed that 53 loci (34.0%) were located in intergenic regions, 74 (47.4%) in intronic regions, and only 2 (1.3%) in exonic regions. Moreover, some SNPs were also found localized in multiple genes. For instance, rs769267 (19p13.11, P.PLACO = 1.41E−09) related to ADHD-ERPBC is mapped to several genes including *SUGP1*, *GATAD2A*, *HAPLN4*, *TM6SF2*, *MAU2*, *TSSK6*, *NDUFA13*, *YJEFN3*, and *CILP2*, in both position and eQTL mapping. Moreover, rs769267 was the only exonic variant among the 13 top SNPs.

**Table 1:** 19 pleiotropic genomic loci identified by FUMA using PLACO results

Trait	Locus	rsID	Cytoband	eQTL support	Functional annotation	Overlap with MAGMA result	CADD	RDB	PP.H4
ADHD-ERNBC	2	rs939584	2p25.3	yes	intergenic	no	2.347	N/A	0.968
ADHD-ERNBC	3	rs28694634	11q13.1	yes	intergenic	yes	1.607	3a	0.898
ADHD-ERNBC	6	rs11882123	19p13.11	yes	downstream	yes	0.354	2b	0.731
ADHD-ERPBC	14	rs28694634	11q13.1	yes	intergenic	yes	1.607	3a	0.946
ADHD-ERPBC	18	rs769267	19p13.11	yes	exonic	yes	9.369	1f	0.783
AN-ERNBC	1	rs112928349	3p21.31	yes	intronic	yes	10.28	6	0.722
ANX-ERNBC	2	rs116459097	4q12	no	ncRNA intronic	no	2.265	7	0.872
ASD-ERPBC	1	rs10197246	2q33.1	yes	intronic	yes	5.378	6	0.787
ASD-ERPBC	11	rs232320	18q11.2	yes	ncRNA intronic	yes	4.662	3a	0.799
BIP-ERPBC	2	rs2647256	4q24	yes	downstream	yes	3.36	N/A	0.916
BIP-ERPBC	8	rs111342015	6p21.1	yes	intronic	no	1.88	5	0.890
DEP-ERNBC	2	rs143582343	4q12	no	ncRNA intronic	no	2.856	6	0.815
DEP-ERNBC	4	rs73035954	7p22.2	yes	intronic	yes	5.572	5	0.826
DEP-ERPBC	21	rs17356521	20q11.21	yes	intronic	yes	4.016	5	0.983
SCZ-ERNBC	5	rs34277109	19p13.11	yes	intergenic	yes	0.166	7	0.908
SCZ-ERPBC	1	rs1451488	2q33.1	yes	intergenic	no	12.94	N/A	0.878
SCZ-ERPBC	5	rs57929572	3p13	no	intronic	yes	8.536	7	0.749
SCZ-ERPBC	12	rs11783093	8p21.1	yes	ncRNA intronic	no	0.044	7	0.726
SCZ-ERPBC	22	rs1469713	19p13.11	yes	intronic	yes	0.175	N/A	0.970

ADHD, attention deficit hyperactivity disorder; AN, anorexia nervosa; ASD, autism spectrum disorder; BIP, bipolar disorder; DEP, depression; SCZ, schizophrenia; ERNBC, estrogen receptor-negative breast cancer; ERPBC, estrogen receptor-positive breast cancer; PP.H4, posterior probability of H4; CADD, The Combined Annotation-Dependent Depletion Score; RDB: RegulomeDB Score.

As a robustness analysis, we applied the condFDR framework to assess cross-trait genetic enrichment between psychiatric disorders and BC. Overall, the condFDR results showed a trend consistent with the primary PLACO analysis. Although locus-level overlap between the two methods was incomplete, genes mapped from condFDR signals, such as *MRTF1*, *FGFR2*, *FURIN*, and *GATAD2A*, were also identified from further gene-level analysis based on PLACO (Table S12).

### Evaluating tissue-specific enrichment

**Figure 3:**
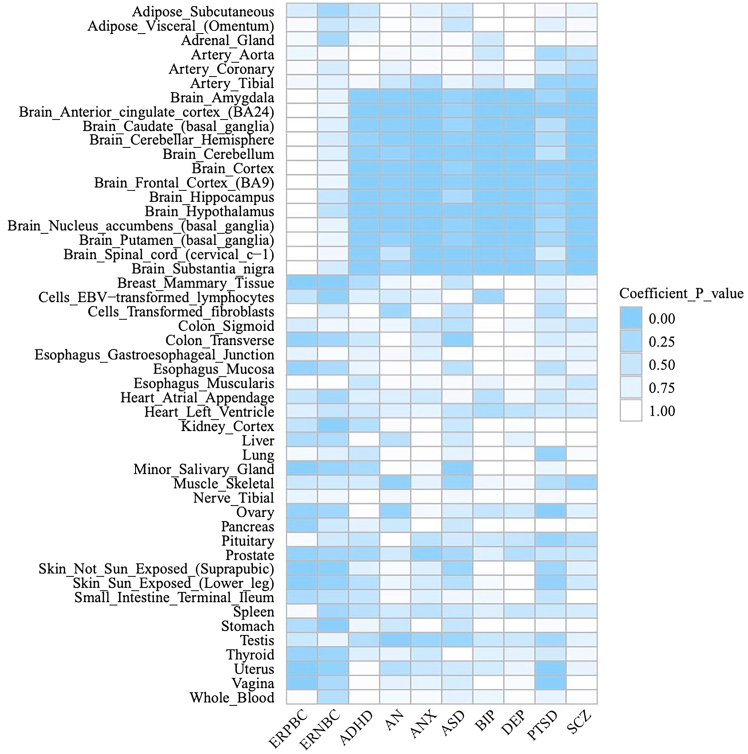
Tissue enrichment analyses Tissue type-specific enrichment of single nucleotide polymorphism (SNP) heritability for breast cancer (BC) and PSY in 49 tissues from GTEx v8 estimated using stratified LDSC applied to specifically expressed genes (LDSC-SEG).

To further identify the expression characteristics of disease-associated variants in BC and psychiatric disorders, tissue-specific enrichment analysis was performed using LDSC-SEG based on GWAS summary statistics (Figure 3; Table S13). It was observed that ERPBC had significant enrichment in breast tissue (FDR <0.05), and there was no significant enrichment for ERNBC. This underscores the tissue consistency of ERPBC, indicating that the genetic structure and biological characteristics of this subtype remain highly consistent. Moreover, psychiatric disorders indicated broad enrichment across various brain tissues. Tissues indicating significant enrichment were selected to further tissue-specific gene-level analyses (Table S14).

### Exploring pleiotropic genes

To prioritize genes genetically implicated in shared susceptibility between BC and psychiatric disorders, we integrated MAGMA, eQTL-informed MAGMA (E-MAGMA), Hi-C-coupled MAGMA (H-MAGMA), and transcriptome-wide association study (TWAS) analyses. The predictive genes were then mapped to functional pathways for further analysis.

**Figure 4:**
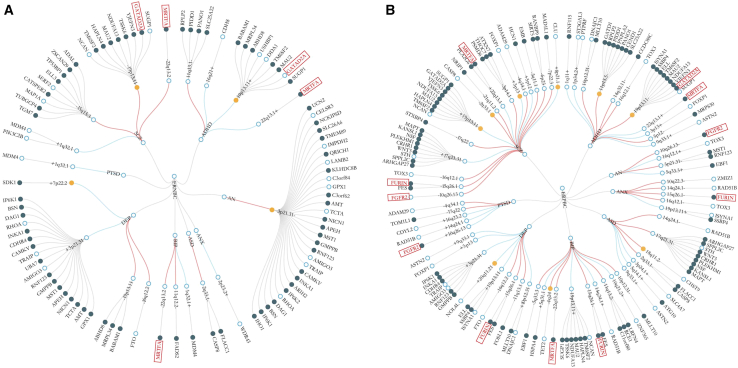
The overall landscape of the pleiotropic associations across breast cancer and eight psychiatric disorders (A) Analysis between ERNBC and PSY. (B) Analysis between ERPBC and PSY. Results of MAGMA analysis on the genes located in or overlapped with the pleiotropic loci based on PLACO outputs. Shared loci identified by colocalization analysis are highlighted in orange; shared loci that showed concordant associations with a certain pair of traits are marked by a plus sign and a blue line; other loci that showed discordant associations are marked by a minus sign and a red line; shared genes identified by E-MAGMA analysis are highlighted in dark blue. Prioritized pleiotropic genes are highlighted.

After Bonferroni correction, MAGMA identified 142 genes overlapping shared loci (Figure 4). Of these, 41 genes had a significant association between multiple trait pairs, with *MRTFA*, *RNF123*, and *FGFR2* being the most enriched. Moreover, 55 (38.73%) genes were novel for psychiatric disorders, and 63 (44.37%) were novel for BC (Tables S11, S15, and S16). After Bonferroni correction, tissue-specific gene analyses using E-MAGMA and TWAS identified 2,038 significant tissue-specific pleiotropic genes (252 unique) (Tables S17 and S18). There were 539 genes enriched by multiple trait pairs, of which 94 genes were significant in MAGMA and E-MAGMA. For instance, DNPH1 was detected in four trait pairs (BIP-ERNBC, BIP-ERPBC, SCZ-ERNBC, and SCZ-ERPBC) and was extensively enriched in different tissues. This study also employed H-MAGMA to identify cell-type-specific risk genes (Tables S14 and S19). MAGMA identified 142 genes, of which 110 were enriched in at least one specific cell type, with MRTFA enriched in 5 different disease pairs, indicating broad cell-specific gene expression. TWAS indicated that SCZ and ERPBC had the highest number of genes from the 27 overlapping genes (*n* = 13). Compared with the TWAS results, 1,671 and 1,348 tissue-specific pleiotropic genes were identified as novel for BC and psychiatric disorders, respectively (Tables S11 and S18). Overall, the results of the tissue-specific gene analysis show a high degree of consistency with the tissue enrichment results in LDSC-SEG. It revealed higher enrichment levels of ERPBC compared with ERNBC in breast tissue, further demonstrating the tissue consistency of ERPBC. As the analysis results indicate, the significant enrichment levels observed across different tissue types suggest that disease-associated variants are more likely to occur near genes expressed in specific tissues. Through the integration of complementary analytical frameworks, *MRTFA*, *FGFR2*, *DNPH1*, *FURIN*, and *GATAD2A* were identified as high-priority pleiotropic candidates, supported not only by their robust convergence across multiple methodologies and trait pairs but also by existing experimental evidence linking them to BC or psychiatric disorders (Table 2).

**Table 2:** 5 prioritized pleiotropic genes identified by multiple level methods and experimental evidence

Gene	Trait_pair (with E RNBC)	Trait pair (with ERPB C)	Experiment al evidence of BC	Experimental evidence of psychiatric disorders	E-MAGMA	H-MAGMA	TWAS	SMR	Biological function
MRTFA	ADHD, BIP, SCZ	ADHD, BIP, SCZ	Jehanno et al.^19^, Jehanno et al.^20^	N/A	yes	yes	yes	no	MRTFA is a transcriptional coactivator of serum response factor, a key regulator of cyto skeleton-related and migration associated gene expression.
FGFR2	N/A	AN, PTSD, SCZ	Tannheimer et al.^21^, Meyer et al.^22^	Carboni et al.^15^, Stevens et al.^16^	yes	no	no	no	FGFR2 encodes a fibroblast growth factor receptor tyrosine kinase that regulates cell proliferation, differentiation, and survival.
DNPH1	BIP, SCZ	N/A	Fugger et al.^23^, Oh et al.^24^	N/A	yes	no	yes	yes	DNPH1 is a nucleotid emetabolism enzyme involved in sanitizing noncanonical nucleotide pools.
FURIN	N/A	ANX, BIP, DEP, SCZ	He et al.^25^, López et al.^26^	Fromer et al.^17^, Hou et al.^18^	yes	yes	yes	no	FURIN is a proprotein convertase that cleaves and activates multi pleprecursor proteins.
GATAD2A	ADHD, SCZ	ADHD, SCZ	Chen et al.^27^	N/A	yes	yes	yes	no	GATAD2A is a core component of the NuR D chromatin-remodeling complex that participates in transcriptional regulation and DNA damage response.

ADHD, attention-deficit/hyperactivity disorder; AN, anorexia nervosa; ANX, anxiety disorder; BIP, bipolar disorder; DEP, depression; SCZ, schizophrenia; E-MAGMA, eQTL-informed multi-marker analysis of GenoMic annotation; ERPBC, estrogen receptor-positive breast cancer; ERNBC, estrogen receptor-negative breast cancer; H-MAGMA, Hi-C-coupled multi-marker analysis of GenoMic annotation; SMR, summary-data-based Mendelian randomization; TWAS, transcriptome-wide association study.

### Pathway level analyses

**Figure 5:**
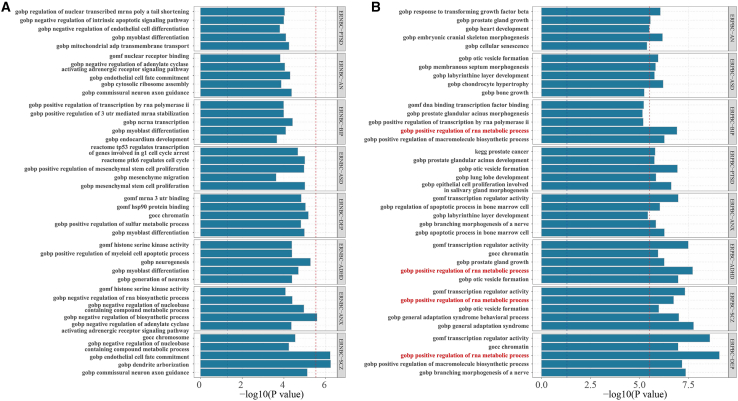
Bar plot showing enrichment of shared pathway between trait pairs in MAGMA gene-set analyses (A) Analysis between ERNBC and PSY. (B) Analysis between ERPBC and PSY. The red line represents the *p* threshold at 1E−05.

To investigate the genetic pathways associated with the discovered pleiotropic genes, various analysis strategies, including gene set analysis performed by MAGMA and pathway enrichment analysis, were employed. After Bonferroni correction and strict adjustment of 9,584 gene sets (from GO_BP, KEGG, and Reactome), it was identified that 12 of 49 pathways were significant and were associated with 5 trait pairs (Figure 5). The positive modulation of RNA metabolism was enriched in most trait pairs (ADHD-ERPBC, BIP-ERPBC, DEP-ERPBC, and SCZ-ERPBC). We also found that the pathway enrichment results exhibited ERPBC specificity with respect to trait pairs, meaning that all identified pathways were enriched in ERPBC-associated trait pairs, highlighting an ERPBC-specific enrichment pattern that may reflect differences in estrogen-responsive transcriptional programs. Importantly, these enrichment results should be interpreted as statistical overrepresentation of genetically implicated genes within predefined pathway annotations, rather than evidence of pathway activation or direct functional involvement.

The Metascape functional annotation platform was employed to enhance the robustness of the results. MAGMA and E-MAGMA analyses identified several over-represented biological processes among the 50 pleiotropic genes in 2 or more trait pairs shared between BC and psychiatric disorders. Significance (FDR <0.05) was observed only for the stem cell proliferation (GO:0072089) and acyltransferase activity (GO:0016746) (Table S20).

### Protein-level SMR analysis

The associations between 1,786 unique proteins and 10 phenotypes were comprehensively assessed using SMR. Then, associations that failed to pass the HEIDI test were eliminated. After multi-SNP-SMR sensitivity analysis and Bonferroni correction, 41 genetically prioritized plasma proteins were retained (Figure S3; Table S21). Among these, 5 proteins were prioritized for BC-related phenotypes, and 36 proteins were prioritized for 6 psychiatric disorder phenotypes, whereas no proteins were prioritized for PTSD or ASD. Because this analysis was based on plasma pQTL data, these results should be interpreted as genetically prioritized protein candidates rather than direct evidence of tissue-specific protein activity in breast or brain tissue.

## Discussion

Although previous studies have preliminarily explored the genetic association between psychiatric disorders and BC, their analytical methods were relatively limited, the spectrum of psychiatric disorders covered was incomplete, and there has been a lack of in-depth analysis of BC subtypes.^14^^,^^28^ By integrating multidimensional genetic data, this study systematically addresses these limitations, establishing the most comprehensive genetic analysis framework to date for the comorbidity of psychiatric disorders and BC. The conceptual advancement of this study lies in the development of a multi-level genetic analysis framework, which strictly controls false-positive results through a stratified screening strategy, thereby significantly enhancing statistical power. Furthermore, the genetic statistical evidence provided by this study suggests that hormone-responsive regulatory pathways and immune dysregulation may be associated with the shared genetic architecture.

LDSC is a comprehensive approach employed to identify genome-wide genetic correlations. Previous studies have reported weak but significant genetic correlations between DEP, SCZ, and BC.^8^^,^^13^ This study validated the results of previous studies and revealed a Bonferroni-corrected significant correlation between ANX and ERNBC. Since LDSC only assesses concordance of effect directions (genetic correlation), the MiXeR method was employed to quantify the number of shared causal variants (genetic overlap), providing a complementary perspective on the genetic architecture between BC and psychiatric disorders. MiXeR analysis showed that compared with the psychiatric disorders, the polygenicity of both BC subtypes is significantly lower, and that the directional effect was consistent with the non-significant genome-wide genetic correlations observed between BC and ADHD, AN, BIP, and PTSD. These findings were further supported by LAVA, which identified genomic regions showing significant local genetic correlation, suggesting that both disease-promoting and protective effects may simultaneously be involved in BC and psychiatric disorders. The literature suggests that there is increased comorbidity between BC and certain psychiatric disorders.^6^^,^^7^^,^^8^^,^^9^^,^^10^^,^^11^ However, these associations can be influenced by external factors such as the use of psychotropic medication and smoking. The current research data are controversial, for instance, a large cohort study found no association between psychotropic drug use and the risk of invasive BC.^29^ However, a 2024 nested case-control study from Sweden revealed that using prolactin-elevating antipsychotics increases the risk of BC.^30^ The findings of the presented study suggest that, in addition to treatment modalities, genetic factors also play a crucial role in the pathogenesis of both diseases.

To investigate the underlying genetic architecture, vertical and horizontal pleiotropy analyses were performed. LCV and LHC-MR approaches were used to evaluate putative genetically inferred directional associations between PTSD and two BC subtypes, as well as between ANX and ERNBC. Overall, the directionality analyses provided limited evidence for vertical pleiotropy. Therefore, the LHC-MR findings should be interpreted as model-dependent and suggestive, rather than as definitive evidence of direct causal effects. This finding is consistent with a previous MR study that did not find a robust association between psychiatric disorders and BC. The authors suggest that some of the significant findings observed may be false positives due to horizontal pleiotropy.^28^ These findings suggest that the shared genetic architecture between BC and psychiatric disorders is more likely to be explained by horizontal pleiotropy than by vertical pleiotropy.

In combination with PLACO, horizontal pleiotropy analysis was also performed to assess the genetic variants associated with psychiatric disorders and BC phenotypes. Under a threshold of PP.H4 > 0.7, ANNOVAR identified 19 high-confidence pleiotropic loci. Of these loci, rs769267 was annotated as an exonic variant in genes such as *GATAD2A*, with a colocalization value of PP.H4 = 0.787. Our study also evaluated several pleiotropic loci in multiple trait pairs. For instance, rs4702, located in the 3′UTR region of *FURIN*, was mapped to the most trait pairs (ANX-ERPBC, BIP-ERPBC, DEP-ERPBC, and SCZ-ERPBC) according to both PLACO and FUMA analyses. In gene-level analysis, *FURIN* was enriched in both the frontal lobe tissue in E-MAGMA and midbrain dopaminergic neurons in H-MAGMA. We employed two strategies to identify pleiotropic genes. MAGMA revealed multiple genes with significant associations that overlapped with FUMA-identified loci. *MRTFA* was identified as a pleiotropic gene across five trait pairs, including ADHD-ERPBC, ADHD-ERNBC, SCZ-ERPBC, SCZ-ERNBC, and BIP-ERPBC. Also, *FGFR2* was identified as a pleiotropic gene associated with ERPBC across AN, PTSD, and SCZ. The E-MAGMA analysis of the ERNBC-SCZ and ERNBC-BIP trait pairs revealed that *DNPH1* was a tissue-specific pleiotropic gene enriched in the caudate nucleus, cerebellar hemisphere, and putamen. The ERPBC analysis showed that the tissue types with specific gene expression included whole blood, the nucleus accumbens, the frontal cortex, and digestive tract-associated tissues. Based on convergence across multiple methods, recurrence across multiple trait pairs, and support from previous experimental evidence, we identified a small set of high-priority pleiotropic candidate genes—*GATAD2A*, *FURIN*, *MRTFA*, *FGFR2*, and *DNPH1*.

A review of prior studies indicates that high-priority pleiotropic candidate genes converge on transcriptional regulation, hormone responsiveness, and immune-related processes. *FURIN* is involved in precursor protein processing, and its dysregulation is associated with altered levels of PD-1 in the immune microenvironment.^31^^,^^32^ It may also influence susceptibility to SCZ through aberrant nerve growth factor processing.^18^ Consistently, *FURIN*-dependent immune regulation has been linked to improved BC clearance, with therapeutic targeting of this pathway showing antitumor potential.^25^^,^^33^ As a key regulator of cytoskeletal signaling, *MRTFA* exhibits aberrant accumulation in ERPBC, which is associated with estrogen-mediated transcriptional repression and hormone escape phenotypes.^19^^,^^20^ Its dysregulation is also implicated in immune dysfunction and may connect shared genetic susceptibility to inflammatory transcriptional programs through gene program reprogramming and neuronal plasticity.^34^^,^^35^^,^^36^ *FGFR2* is a well-established BC susceptibility gene, whose aberrant activation is linked to STAT3 and ERK-YY1 signaling as well as PD-L1-mediated immune evasion.^22^^,^^37^^,^^38^^,^^39^ Targeting FGFR2-related pathways (e.g., bevacizumab) has demonstrated antitumor efficacy via modulation of signaling and immune responses.^40^ Notably, enrichment of *FGFR2* variants in psychiatric disorders suggests a potential link to fibroblast growth factor regulatory networks.^41^ Our findings corroborate previous studies and further support a pleiotropic role for *GATAD2A* in BC and ADHD.^13^ *GATAD2A*-associated signal transduction and transcriptional programs are closely related to tumor progression.^27^^,^^42^ In addition, the DNPH1 enzyme has been identified as a tumor-promoting factor in BC and a potential therapeutic target for BIP.^24^^,^^43^ However, the neurobiological mechanisms linking these two genes to their roles in psychiatric disorders remain unclear. Overall, immune regulation represents a plausible mechanistic framework between psychiatric disorders and BC. However, current evidence is largely based on integrative analyses and requires further experimental validation.

Functional exploration of pathway analysis has revealed several genetically associated pathways, such as the positive regulation of RNA metabolism and acyltransferase activity. One study found that carnitine palmitoyltransferase 1 (CPT1) enhances the response to HER2-targeted therapy by promoting fatty acid oxidation.^44^ Concurrently, this enzyme has also been linked to cognitive impairment and motor dysfunction.^45^ Although the biological mechanisms underlying the association between acyltransferase activity and brain dysfunction and BC remain unclear, our study further supports genetic association between this pathway and both diseases. Positive regulation of RNA metabolism was specifically enriched in ERPBC-associated trait pairs, suggesting that genes contributing to shared susceptibility were overrepresented in RNA metabolism-related pathways. An important and potentially novel observation of this study is that molecular convergence and pathway enrichment were stronger for ERPBC than for ERNBC. This ERPBC-specific pattern, together with breast tissue enrichment, suggests that shared disease-associated variants may preferentially converge on regulatory genomic regions or genes involved in hormone-responsive transcriptional programs. In ERPBC, ER signaling operates through an estrogen-responsive transcriptional network involving ESR1/ERα and cooperating factors such as *FOXA1* and *GATA3*, which together shape enhancer usage, chromatin accessibility, and transcriptional programs.^46^^,^^47^^,^^48^ Therefore, the present findings may be more appropriately interpreted as reflecting a shared hormone-responsive regulatory context. This interpretation is also biologically plausible in psychiatric disorders, where estrogen-responsive programs have been implicated in neurotransmitter regulation, and psychosis-related vulnerability.^49^^,^^50^^,^^51^ However, our enrichment results only indicate statistical overrepresentation of genetically implicated genes, and further validation through functional experiments is required.

### Limitations of the study

This study investigated the shared genetic architecture between psychiatric disorders and BC. However, there are several potential limitations. First, like many other studies, the study analyzed summary-level data rather than individual-level data. Restricted access to individual-level data and the lack of a substantial cohort precluded further stratification by factors such as sex, age, or other demographic characteristics. In particular, psychiatric disorder GWAS datasets included both sexes, while BC samples are female-only. Second, the analysis was limited to the European population only. Thus, the findings are not generalizable to other ancestries. Differences in LD structure, allele frequencies, and regulatory architecture across ancestries may alter the detectability of pleiotropic loci and the interpretation of colocalization and gene-mapping results. Future multi-ancestry validation studies are needed to determine whether the prioritized loci, genes, proteins, and pathway-level enrichment patterns are transferable across populations. Third, the results only identified common variants, whereas rare variants also play a crucial role.^52^^,^^53^ Fourth, our study is limited to autosomal analyses and does not include the X and Y chromosomes. Future studies could consider targeted analyses of the X and Y chromosomes to better understand sex-specific genetic effects. Fifth, no experimental validation is provided in this study. Experimental validation using relevant BC or neural cell models will be an important future direction to clarify their mechanistic relevance. Lastly, the sample size of studied traits was relatively small, which may have limited statistical power; therefore, results should be interpreted with caution.

## Resource availability

### Lead contact

Requests for further information and resources should be directed to and will be fulfilled by the lead contact, Hongyan Jia (swallow_jhy@163.com).

### Materials availability

This study did not generate new, unique reagents.

### Data and code availability

• This paper analyzes existing, publicly available datasets. Details of all datasets, including accession codes and dataset identifiers, are provided in Table S1 and the key resources table.
• Custom code used for the analyses in this study is publicly available at GitHub: https://github.com/WccCccZ/shared-genetics-of-BC-PSY. All software and algorithms used in this study, including LDSC, MiXeR, LAVA, LCV, LHC-MR, PLACO, FUMA, HyPrColoc, MAGMA, e-MAGMA, TWAS (FUSION), SMR, COLOC, pleioFDR, and R, are listed in the key resources table.
• Any additional information required to re-analyze the data reported in this paper is available from the lead contact upon reasonable request.

## Acknowledgments

This study was supported by the Central Guiding Local Science and Technology Development Special Project (grant no. YDZJSX2024D068), the 10.13039/501100004480Natural Science Foundation of Shanxi Province (grant no. 202303021222339), Shanxi Province Higher Education “Billion Project” Science and Technology Guidance Project and MOE key Laboratory of Coal Environmental Pathogenicity and Prevention (grant no. 2C622024113), and 10.13039/501100003398Shanxi Scholarship Council of China (grant no. 2022-119). We would like to thank the authors of all the GWAS who made their summary statistics available for the benefit of this study, including the following: the BCAC, IEU OpenGWAS, and the PGC without whom this effort would not be possible.

## Author contributions

Conceptualization, H.J.; funding acquisition, H.J., J.L., and Y.W.; supervision, H.J. and Y.W.; project administration, Y.W.; data curation, C.W. and Y.Z.; software, C.W., J.H., and K.H.; methodology, J.H. and L.L.; validation, Y.L. and J.L.; writing – original draft, X.S., X.W., and J.W.; and writing – review and editing, all authors.

## Declaration of interests

The authors declare no competing interests.

## STAR★Methods

### Key resources table

**Table:** 

REAGENT or RESOURCE	SOURCE	IDENTIFIER
**Deposited data**		

Attention deficit hyperactivity disorder (ADHD)	Demontis et al.^54^	https://figshare.com/articles/dataset/adhd2022/22564390
Anorexia nervosa (AN)	Watson et al.^55^	https://figshare.com/ndownloader/files/28169271/pgcAN2.2019-07.vcf.tsv.gz
Anxiety disorders GWAS summary statistics (ANX)	Li et al.^56^	https://figshare.com/articles/dataset/Anxiety-Disorders-plink_meta_P_Sorted_gz/23659653/1
Autism spectrum disorder GWAS summary statistics (ASD)	Grove et al.^57^	https://figshare.com/ndownloader/files/28169292/iPSYCH-PGC_ASD_Nov2017.gz
Bipolar disorder GWAS summary statistics (BIP)	Mullins et al.^58^	https://gwas.mrcieu.ac.uk/datasets/ieu-b-5110/
Depression GWAS summary statistics (DEP)	Als et al.^59^	https://ipsych.dk/en/research/downloads/
Post-traumatic stress disorder (PTSD)	Nievergelt et al.^60^	https://figshare.com/articles/dataset/ptsd2019/14672133
Schizophrenia (SCZ)	Trubetskoy et al.^61^	https://figshare.com/articles/dataset/scz2022/19426775
ER-negative breast cancer (ERNBC)	Michailidou et al.^62^	https://gwas.mrcieu.ac.uk/datasets/ieu-b-1128/
ER-positive breast cancer (ERPBC)	Michailidou et al.^62^	https://gwas.mrcieu.ac.uk/datasets/ieu-b-1127/

**Software and algorithms**		

LDSC	Bulik-Sullivan et al.^63^	https://github.com/bulik/ldsc
MiXeR	Frei et al.^64^	https://github.com/precimed/mixer
LAVA	Werme et al.^65^	https://github.com/josefin-werme/LAVA
LCV	O'Connor & Price et al.^66^	https://github.com/lukejoconnor/LCV
lhcMR	Darrous et al.^67^	https://github.com/LizaDarrous/lhcMR
PLACO	Ray et al.^68^	https://github.com/RayDebashree/PLACO
FUMA	Watanabe et al.^69^	http://fuma.ctglab.nl/
HyPrColoc	Foley et al.^70^	https://github.com/jrs95/hyprcoloc
MAGMA	de Leeuw et al.^71^	https://ctg.cncr.nl/software/magma
e-MAGMA	Gerring et al.^72^	https://github.com/eskederks/eMAGMA-tutorial
TWAS (FUSION)	Gusev et al.^73^	http://gusevlab.org/projects/fusion/
SMR	Zhu et al.^74^	https://yanglab.westlake.edu.cn/software/smr/
COLOC	Giambartolomei et al.^75^	https://github.com/chr1swallace/coloc
pleioFDR	Andreassen et al.^76^	https://github.com/precimed/pleiofdr
R	R Foundation for Statistical Computing	https://www.r-project.org/

### Method details

#### Data sources and quality control

Publicly available GWAS summary statistics for psychiatric disorders from the Psychiatric Genomics Consortium (PGC) and GWAS summary statistics for BC from the Breast Cancer Association Consortium (BCAC) were analyzed in this study. From a GWAS meta-analysis, the ERNBC dataset, including 127,442 individuals (21,468 cases and 105,974 controls), and the ERPBC dataset comprising 175,475 individuals (69,501 cases and 105,974 controls) were acquired.^62^ All GWAS data in this study are derived from European samples. European ancestry samples were defined based on the original studies’ quality control procedures, which applied genetic principal component analysis to exclude non-European individuals. For some psychiatric disorder cohorts, ancestry was also supported by self-reported information; details are provided in the original publications.^54^^,^^55^^,^^56^^,^^57^^,^^58^^,^^59^^,^^60^^,^^61^ Genotype imputation and imputation-quality filtering were performed in the original GWAS studies. For downstream analyses, summary statistics were harmonized to autosomal variants with valid rsIDs. Variants located on sex chromosomes, duplicated variants, variants with missing rsIDs, variants with MAF < 0.01, and variants in the major histocompatibility complex region (chr 6: 25-35 Mb) were excluded. No additional global LD pruning or clumping was applied before the primary analyses because the approaches in this study either use genome-wide SNP information or account for LD through external LD reference panels. In this study, 1000 Genomes Project v3 was used as a reference panel for European samples, and the MHC region was excluded. The detailed information on the databases and the included GWAS data are summarized in Table S1.

#### Investigating shared genetic architecture

In this study, four complementary approaches were applied to dissect the shared genetic architecture between psychiatric disorders and BC: (i) Genetic correlation (estimated by LDSC and LAVA) reflects the concordance of effect directions across traits; (ii) Genetic overlap (estimated by MiXeR) quantifies the number of shared causal variants, regardless of direction; (iii) Vertical pleiotropy refers to genetic variants influencing one trait that causally affects another (estimated by LCV and LHC-MR); (iv) Horizontal pleiotropy (estimated by PLACO) refers to genetic variants that independently affect multiple traits. These measures altogether capture different aspects of shared genetic architecture.

#### Genetic correlation

Linkage disequilibrium score regression (LDSC) is the approach that quantifies the heritability of each trait and genetic correlation between psychiatric disorders and BC.^63^^,^^77^ In LDSC, the standard error estimated by the leave-one-out method identified the reliability of the correlation estimates. In this study, first, the univariate LDSC method was employed to analyze the input GWAS summary statistics for examining the polygenic SNP heritability (h2SNP). Then the bivariate LDSC method was utilized. All tests conducted in the bivariate LDSC were two-sided, and significant data were acquired after adjusting for multiple tests for interpretation. We used the LDSC intercept to evaluate potential sample overlap between GWAS datasets. In our analysis, intercepts between BC and psychiatric disorders datasets indicated no substantial sample overlap, supporting the reliability of this study. LD scores for LDSC were obtained from the official precomputed European LD score files distributed with LDSC, which were derived from the 1000 Genomes European reference panel (https://github.com/bulik/ldsc).

#### Local genetic correlation

The local genetic correlation among semi-independent genomic loci was assessed via Local Analysis of [co] Variant Annotation (LAVA). LAVA employs a fixed-effects statistical model to estimate local SNP heritability and genetic correlations (loc-rgs), providing insight into shared genetic architecture at a locus-specific level.^65^ In total, 2,495 genomic regions were targeted, which were designed to minimize LD while maintaining approximately equal sizes of around 1 Mb. Local genetic correlations were assessed only for loci indicating nominally significant heritability in both traits (p < 1E-4). For each analyzed trait pair, the *p*-values were adjusted by correcting for False Discovery Rate (FDR).

#### Genetic overlap

To investigate the genetic overlap between traits, MiXeR was applied. It is a statistical tool designed to distinguish shared and unique genetic architectures using GWAS summary statistics.^64^^,^^78^ The model assumes that SNP effects arise from a two-component mixture consisting of non-causal variants with zero effects and causal variants with normally distributed non-zero effects. In the MiXeR analysis, the first step is to compute univariate estimates for each phenotype, assuming that SNP effects follow a mixture of two variants (causal and non-causal). The analysis can evaluate polygenicity (π, the proportion of causal variants), discoverability (σ2, the variance of effect sizes per causal variant), and SNP-based heritability for each trait. For better interpretation, polygenicity was delineated as the number of causal variants with the most significant effects required to account for 90% of the SNP heritability (h^2^).

The next step of MiXeR is to build a bivariate causal mixture model to estimate the total number of shared causal variants between the two traits and the number of phenotype-specific causal variants and non-causal variants. Furthermore, shared polygenicity (the number of overlapping causal variants) and the correlation of their effect sizes were estimated to identify the magnitude and directionality of genetic overlap. Model reliability was evaluated using repeated parameter optimization and comparison of the best-fitting model against constrained reference models representing minimum and maximum genetic overlap. Convergence was assessed by stability of parameter estimates across optimization runs and by positive Akaike Information Criterion (AIC) differences between the best-fitting model and the reference models.

#### Genetically inferred directionality analyses

To evaluate putative genetically inferred directional associations underlying the genetic correlations between psychiatric disorders and BC, latent causal variable (LCV) analysis was carried out. The LCV model determines the causal component of genetic correlation between phenotypes by positing a latent variable that exerts directional effects on each trait. While robust, the LCV framework assumes that a single, unidirectional latent factor drives genetic correlation, which may be confounded by bidirectional effects or multiple latent influences. To address this limitation, LHC-MR, an advanced MR framework, was employed.^67^ LHC-MR was used to infer bidirectional associations between complex traits. LHC-MR improves traditional MR methods by modeling an unobserved heritable confounder that influences both exposure and outcome traits, thus preserving the critical exchangeability assumption even in sample overlap. LHC-MR provides more accurate causal effect estimates than conventional approaches by jointly estimating direct, indirect, and null SNP effects on each trait. Statistical significance was determined using a Bonferroni-adjusted threshold.

To assess the robustness of the directional inference results, we additionally summarized the conventional two-sample Mendelian randomization (MR) estimates generated within the implemented LHC-MR workflow. These complementary analyses included the inverse-variance weighted (IVW), MR-Egger, and weighted median methods based on SNP instruments. Concordance in the direction and magnitude of effect estimates across methods was considered supportive of a more robust directional association, whereas inconsistent results were interpreted with caution. Instrument validity and model robustness were further evaluated by examining instrument strength, heterogeneity, and directional pleiotropy statistics, including F statistics, Cochran’s Q, and the MR-Egger intercept. For Cochran’s Q-statistic, significant heterogeneity was indicated if *p* < 0.05; F-statistic was calculated to estimate the weak instrumental bias, with F < 10 considered to indicate suspected bias.

#### Cross-trait meta-analysis

Cross-trait analysis was carried out to assess horizontal pleiotropic variants. PLACO was carried out to determine significant SNPs shared between BC and psychiatric disorders, based on GWAS summary statistics. The compound null hypothesis stated that the variants are associated with none or only one trait.^68^ Pleiotropic variants were considered significant if they exceeded the genome-wide significance level threshold (*p* < 5.00E-08). Z-scores for the two traits at a given genetic variant were set as the test statistic to evaluate the hypothesis.

To improve the robustness of the analysis, we applied the conditional false discovery rate (condFDR) method to improve the detection of shared genetic signals. This approach is based on an empirical Bayesian framework and estimates the posterior probability that a given SNP is null for the target phenotype while incorporating cross-trait association information from GWAS summary statistics. By leveraging information across phenotypes, condFDR re-ranks SNP-level association evidence and prioritizes variants showing stronger evidence of association across multiple traits, thereby increasing statistical power for locus discovery.

#### Functional Mapping and annotation

For the pleiotropic SNPs assessed by PLACO, the Functional Mapping and Annotation of Genetic Associations (FUMA) was performed to characterize significant loci, annotate the potential effects of the risk loci, and conduct functional gene mapping.^69^ The lead SNPs and those in LD were then mapped to genes based on their functional properties using Annotate Variation (ANNOVAR).^79^^,^^80^^,^^81^^,^^82^ Moreover, FUMA allows the mapping of genomic risk loci to the appropriate genes, using positional mapping, expression quantitative trait loci (eQTL) mapping, and chromatin interaction mapping.^75^

#### Colocalization

Bayesian colocalization analysis was performed on the pleiotropic loci identified by FUMA. The results can help identify potential causal variants among the pleiotropic loci.^83^ Colocalization was conducted using the coloc framework under five mutually exclusive hypotheses: no association with either trait (H0), association with trait 1 only (H1), association with trait 2 only (H2), association with both traits driven by distinct causal variants (H3), and association with both traits driven by a shared causal variant (H4). The default coloc priors were used: p1 = 1 × 10-4, p2 = 1E-4, and p12 = 1E-5. This study employed the posterior probability of H4 (PP.H4 > 0.7) from the Bayesian colocalization algorithms to identify genetic loci with evidence of colocalization.

#### Tissue enrichment

LDSC for specific gene expression (LDSC-SEG) was performed to evaluate tissue-specific heritability enrichment across different diseases, and certain tissues were selected based on their enrichment levels for subsequent tissue-specific gene-level analysis.^84^ For this analysis, the GWAS summary statistics, tissue-specific gene expression data from the Genotype-Tissue Expression (GTEx) project (v8, 49 tissues), and Franke lab datasets (152 cell types) were used.^85^^,^^86^

#### Gene level analyses

Based on PLACO results and single-trait GWAS summary statistics, Gene-level and gene-set analyses were conducted using MAGMA.^71^ This regression-based framework aggregates SNP associations while adjusting for LD and gene size. Gene-based associations were assessed using a principal components regression model, incorporating LD reference data from the 1000 Genomes Project European population.^87^ Significance thresholds were identified by Bonferroni correction to account for multiple tests.

The limitations in assigning non-coding SNPs to functionally relevant genes were addressed using two extensions of MAGMA: E-MAGMA (eQTL-informed MAGMA)^72^ and H-MAGMA (Hi-C-coupled MAGMA).^88^ Similarly, transcriptome-wide association studies (TWAS) was applied in this study to investigate tissue specificity. For TWAS FUSION analysis, the tissues used for E-MAGMA were employed.^73^

#### Pathway level analyses

To explore the putative biological implications of pleiotropic genes, gene set analyses, including Gene Ontology (GO) gene sets and Kyoto Encyclopedia of Genes and Genomes (KEGG), were carried out based on the MsigDB dataset^89^ and MAGMA-based MAGMA results. These analyses identified pathways enriched for genes indicating significant associations with the phenotype of interest. A Bonferroni correction was applied to account for multiple tests.

Subsequently, the significant overlapping genes identified in MAGMA and E-MAGMA analyses were submitted to functional enrichment analysis via the Metascape platform.^90^^,^^91^

#### pQTL-based SMR analysis

A pQTL-based SMR analysis was carried out using genome-wide significant cis-pQTLs as instrumental variables to elucidate the association between protein abundance and disease phenotypes.^74^ Cis-pQTLs were defined as SNPs within a 1 Mb window of the corresponding protein-coding gene's transcription start site. The analysis was predicated on cis-pQTLs sourced from the UK Biobank Pharma Proteomics Project (UKB-PPP). This project is a comprehensive population-based resource comprising plasma samples from 34,557 individuals of European descent. The cohort underwent large-scale, high-throughput proteomic analysis of 2,940 plasma proteins using the OlinkExplore platform. However, it should be emphasized that analyses based on plasma protein levels may not reflect protein activity in breast or brain tissue. The SNPs associated with protein abundance indicating the Bonferroni-corrected significance threshold (*p* < 2.80E-05) were included. Potential associations may arise from two mechanisms: (1) pleiotropy, in which a single causal variant simultaneously regulates both protein abundance and the outcome trait; (2) linkage, in which two independent causal variants, strongly linked by linkage disequilibrium (LD), independently influence protein abundance and the outcome trait, respectively. To distinguish between these two mechanisms, we employed the Heterogeneity in Dependent Instruments (HEIDI) test.^92^ This test evaluates the heterogeneity of dependence among instrumental variables to test the null hypothesis that the association is driven by a single causal variant. When the *p*-value of the HEIDI test statistic is greater than 0.01, the null hypothesis cannot be rejected, supporting the possibility that the pleiotropy mechanism is at play. Furthermore, sensitivity analyses were performed using a multi-SNP SMR approach to increase the robustness, with significance defined as p < 0.05.

### Quantification and statistical analysis

All analyses were conducted using publicly available GWAS summary statistics. Sample sizes for each dataset, including the numbers of cases and controls where applicable, are provided in Table S1 and the key resources table. In this study, n represents the number of individuals included in the original GWAS datasets or the number of genetic variants, loci, genes, proteins, or pathways analyzed, as specified in the corresponding Results sections, figure legends, and supplementary tables.

Genome-wide genetic correlation was assessed using LDSC, and nominal significance was defined as *p* < 0.05. Bonferroni correction was applied where multiple trait-pair tests were performed. Local genetic correlations were estimated using LAVA, and false discovery rate correction was applied for multiple local tests. MiXeR model fit was evaluated using AIC-based comparisons. Directional genetic associations were evaluated using LCV and LHC-MR, with Bonferroni-adjusted significance thresholds used for multiple testing. Conventional MR sensitivity analyses included inverse-variance weighted, MR-Egger, and weighted median methods, with Cochran’s Q statistic, MR-Egger intercept, and F statistics used to assess heterogeneity, directional pleiotropy, and weak-instrument bias.

Cross-trait pleiotropic SNPs were identified using PLACO at a genome-wide significance threshold of p < 5E−8. Genomic loci were annotated using FUMA and ANNOVAR. Bayesian colocalization was performed using coloc, and loci with PP.H4 > 0.7 were considered to show evidence of colocalization. Gene-level and gene-set analyses were performed using MAGMA, E-MAGMA, H-MAGMA, and TWAS/FUSION, with Bonferroni correction applied where appropriate. Pathway enrichment analyses were performed using MAGMA and Metascape. Protein-level associations were assessed using SMR, followed by HEIDI filtering and multi-SNP SMR sensitivity analyses. Unless otherwise specified, all statistical tests were two-sided.

All software and algorithms used in the analyses, together with versions and resource links, are listed in the key resources table.

## Supplemental information

Document S1. Figures S1–S3

Table S1. Overview of eight psychiatric disorders and two subtypes of breast cancer included in this study

Table S2. Univariate and bivariate LDSC results for eight psychiatric disorders and two subtypes of breast cancer

Table S3. Univariate and bivariate MiXeR results for eight psychiatric disorders and two subtypes of breast cancer

Table S4. Univariate LAVA results for each eight psychiatric disorders and two subtypes of breast cancer

Table S5. Bivariate LAVA results between eight psychiatric disorders and two subtypes of breast cancer

Table S6. Causal relationship between eight psychiatric disorders and two subtypes of breast cancer

Table S7. Genomic risk loci identified for results of eight psychiatric disorders and two subtypes of breast cancer using FUMA

Table S8. Genomic risk loci identified for single trait GWAS results of eight psychiatric disorders and two subtypes of breast cancer using FUMA

Table S9. Prioritized genes identified for PLACO results of eight psychiatric disorders and two subtypes of breast cancer by functional annotation and mapping of genomic risk loci using FUMA

Table S10. Prioritized genes identified for single trait GWAS results of eight psychiatric disorders and two subtypes of breast cancer by functional annotation and mapping of genomic risk loci using FUMA

Table S11. Candidate pleiotropic genes identified for PLACO results of eight psychiatric disorders and two subtypes of breast cancer using MAGMA and TWAS

Table S12. Genomic risk loci identified for results of eight psychiatric disorders and two subtypes of breast cancer using FUMA

Table S13. S-LDSC-based heritability enrichment estimates in 49 tissue types or 489 functional categories for PLACO results between eight psychiatric disorders and two subtypes of breast cancer using LDSC-SEG

Table S14. All tissue and cell types selected in this study

Table S15. Gene-based analysis for all genes that were nominally significant for PLACO results between eight psychiatric disorders and two subtypes of breast cancer

Table S16. Gene-based analysis for all genes that were nominally significant for single trait GWAS results of eight psychiatric disorders and two subtypes of breast cancer

Table S17. E-MAGMA analysis for 29 related tissue-specific genes that were nominally significant for PLACO results between psychiatric disorders and two subtypes of breast cancer

Table S18. Transcriptome-wide association study analysis for 10 related tissue-specific genes that were nominally significant for single trait GWAS results of eight psychiatric disorders and two subtypes of breast cancer

Table S19. H-MAGMA analysis for 6 related cell-specific genes that were nominally significant for PLACO results between eight psychiatric disorders and two subtypes of breast cancer

Table S20. Biological processes and enriched signaling pathways between eight psychiatric disorders and two subtypes of breast cancer

Table S21. Proteome-wide MR analysis for causalities of plasma protein levels with eight psychiatric disorders and two subtypes of breast cancer including sensitivity analysis
