# Supplementary material for: Cross-trait genetic architecture between breast cancer and psychiatric disorders
Source: iScience. 2026 Jul 13;29(8):116767. doi: 10.1016/j.isci.2026.116767 (PMC13382130; doi:10.1016/j.isci.2026.116767)

iScience, Volume *29*

Supplemental information

Cross-trait genetic architecture

between breast cancer and psychiatric disorders

Canzhou Wang, Jingxi Hu, Yan Lei, Jing Li, Yaochen Zhang, Kai Hu, Liu Liu, Xin Su, Xinxu Wang, Jinqi Wang, Yanhong Wang, and Hongyan Jia

Figure S1: *Bivariate MiXeR Venn diagrams, conditional QQ plots and log-likelihood plots for breast cancer and psychiatric disorder trait pairs.* ***Left panel:*** *Venn diagrams showing the estimated numbers of non-zero variants. The size of the circles represent polygenicity. The estimated genetic correlation is displayed below.* ***Middle panels:*** *Conditional QQ plots illustrating the enrichment of significant SNPs under different P-value thresholds for the complementary trait (0.001, 0.01, 0.1, and all SNPs). Null model curves (black dashed lines) indicate the theoretical -log10(p) distribution under the assumption of no shared causal variants, serving as a baseline. Maximum overlap model curves represent the theoretical upper limit of genetic sharing between the two traits, constrained by the maximal number of shared causal variants. Deviation of the observed curves from the null model indicates statistical support for shared causal variants. Panels involving PTSD are flagged as less reliable due to poorer model fit.* ***Right panel:*** *Log-likelihood plots showing the adjusted negative log-likelihood (-log(L) + const) as a function of the number of shared causal variants (k). Best-fit MiXeR model (blue solid line) represents the maximum likelihood estimate of the negative log-likelihood.*

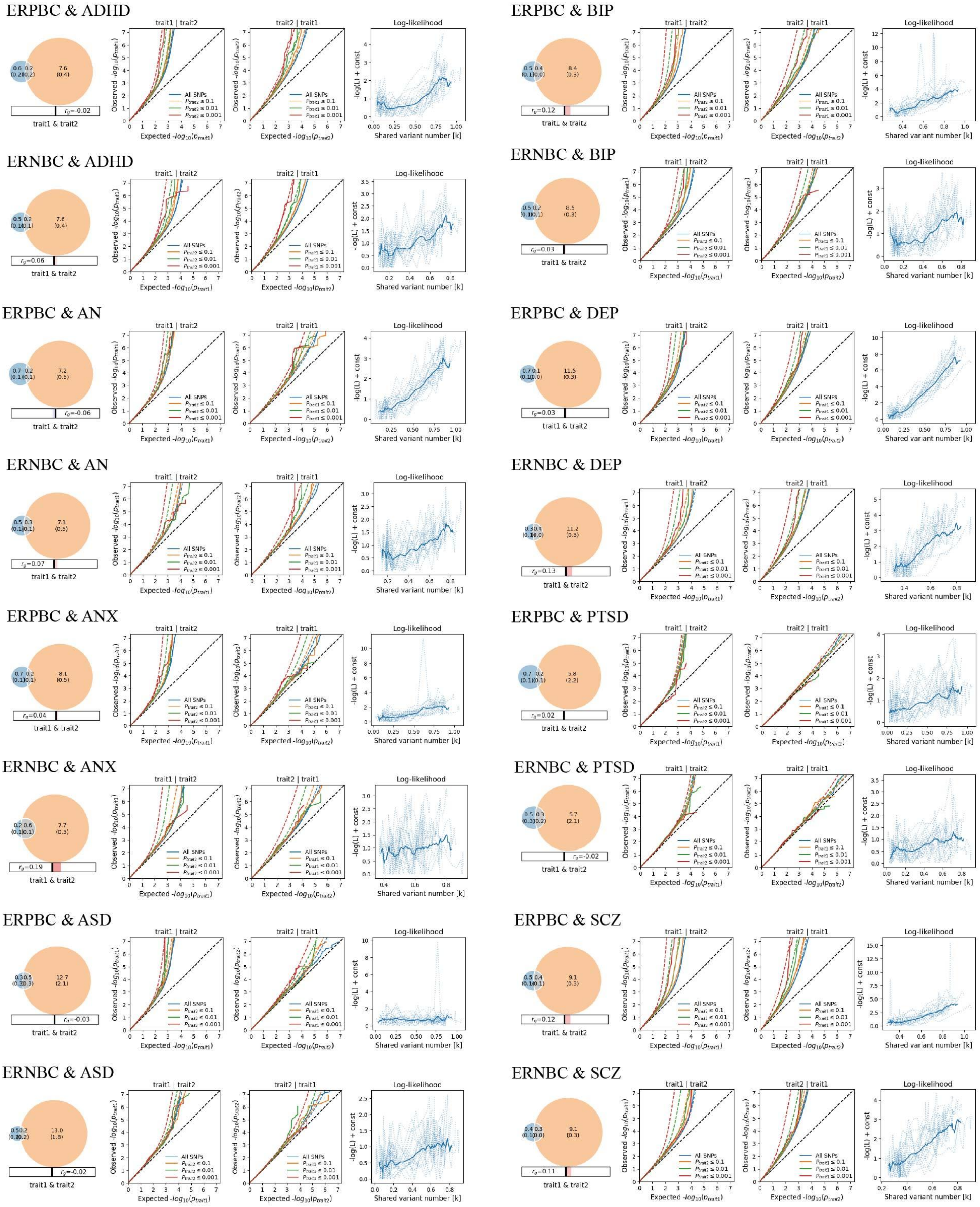
ERPBC & ADHD
rg=-0.02
ERPBC & BIP
rg=0.12
ERNBC & ADHD
rg=0.06
ERNBC & BIP
rg=0.03
ERPBC & AN
rg=-0.06
ERPBC & DEP
rg=0.03
ERNBC & AN
rg=0.07
ERNBC & DEP
rg=0.13
ERPBC & ANX
rg=0.04
ERPBC & PTSD
rg=0.02
ERNBC & ANX
rg=0.19
ERNBC & PTSD
rg=-0.02
ERPBC & ASD
rg=-0.03
ERPBC & SCZ
rg=0.12
ERNBC & ASD
rg=-0.02
ERNBC & SCZ
rg=0.11
trait1 & trait2
trait1 | trait2
trait2 | trait1
Log-likelihood
Shared variant number [k]
-log(L) + const
All SNPs

Figure S2. *Forest plot of genetically inferred directional associations between psychiatric disorders and breast cancer across different Mendelian randomization methods. The figure illustrates suggestive and model-dependent associations rather than definitive causal effects. These estimates may be sensitive to residual pleiotropy, instrument validity, and model assumptions, and should therefore be interpreted with caution.*

outcome	exposure	nsnp	Method	pval	OR(95% CI)
ERPBC	ADHD	21	Inverse variance weighted	0.775	1.018 (0.895 to 1.141)
		21	Weighted median	0.432	0.954 (0.838 to 1.071)
		21	MR Egger	0.248	0.528 (−0.522 to 1.578)
	AN	8	Inverse variance weighted	0.961	1.002 (0.927 to 1.077)
		8	Weighted median	0.680	0.980 (0.881 to 1.078)
		8	MR Egger	0.546	0.810 (0.162 to 1.457)
	ANX	9	Inverse variance weighted	0.199	1.195 (0.923 to 1.467)
		9	Weighted median	0.568	1.075 (0.826 to 1.325)
		9	MR Egger	0.822	1.476 (−1.784 to 4.736)
	ASD	2	Inverse variance weighted	0.339	1.061 (0.940 to 1.183)
	BIP	44	Inverse variance weighted	0.754	0.984 (0.885 to 1.084)
		44	Weighted median	0.168	0.930 (0.826 to 1.034)
		44	MR Egger	0.093	2.038 (1.225 to 2.852)
	DEP	115	Inverse variance weighted	0.540	1.030 (0.935 to 1.125)
		115	Weighted median	0.748	0.982 (0.874 to 1.091)
		115	MR Egger	0.295	1.421 (0.766 to 2.076)
	PTSD	1	Wald ratio	0.282	0.822 (0.466 to 1.178)
	SCZ	122	Inverse variance weighted	<0.001	1.092 (1.054 to 1.129)
		122	Weighted median	0.031	1.042 (1.005 to 1.080)
		122	MR Egger	0.002	1.382 (1.177 to 1.588)

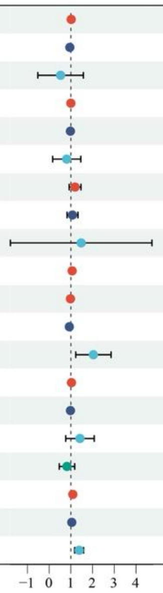

outcome	exposure	nsnp	Method	pval	OR(95% CI)
ERNBC	ADHD	21	Inverse variance weighted	0.939	1.005 (0.886 to 1.123)
		21	Weighted median	0.753	1.021 (0.894 to 1.147)
		21	MR Egger	0.986	1.010 (−0.045 to 2.065)
	AN	8	Inverse variance weighted	0.680	0.971 (0.833 to 1.110)
		8	Weighted median	1.000	1.000 (0.868 to 1.132)
		8	MR Egger	0.012	0.279 (−0.430 to 0.987)
	ANX	9	Inverse variance weighted	0.244	1.155 (0.912 to 1.398)
		9	Weighted median	0.183	1.217 (0.928 to 1.507)
		9	MR Egger	0.753	1.626 (−1.281 to 4.533)
	ASD	2	Inverse variance weighted	0.132	1.116 (0.973 to 1.258)
	BIP	44	Inverse variance weighted	0.274	0.942 (0.836 to 1.049)
		44	Weighted median	0.080	0.889 (0.757 to 1.021)
		44	MR Egger	0.739	1.166 (0.270 to 2.062)
	DEP	115	Inverse variance weighted	0.043	1.093 (1.007 to 1.178)
		115	Weighted median	0.041	1.136 (1.013 to 1.258)
		115	MR Egger	0.294	1.375 (0.783 to 1.967)
	PTSD	1	Wald ratio	0.383	1.204 (0.786 to 1.622)
	SCZ	122	Inverse variance weighted	0.021	1.040 (1.007 to 1.074)
		122	Weighted median	0.260	1.023 (0.983 to 1.063)
		122	MR Egger	0.040	1.220 (1.032 to 1.408)

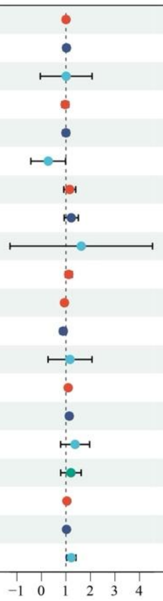

Figure S3. *Volcano plots derived from the SMR analysis illustrate the associations between circulating protein levels and each trait, with effect estimates shown on the x-axis and corresponding P values on the y-axis. Red dots denote significant positive associations, blue dots denote significant negative associations, and gray dots indicate non-significant associations. As one of high-priority pleiotropic candidate genes, DNPH1 is marked in this figure.*

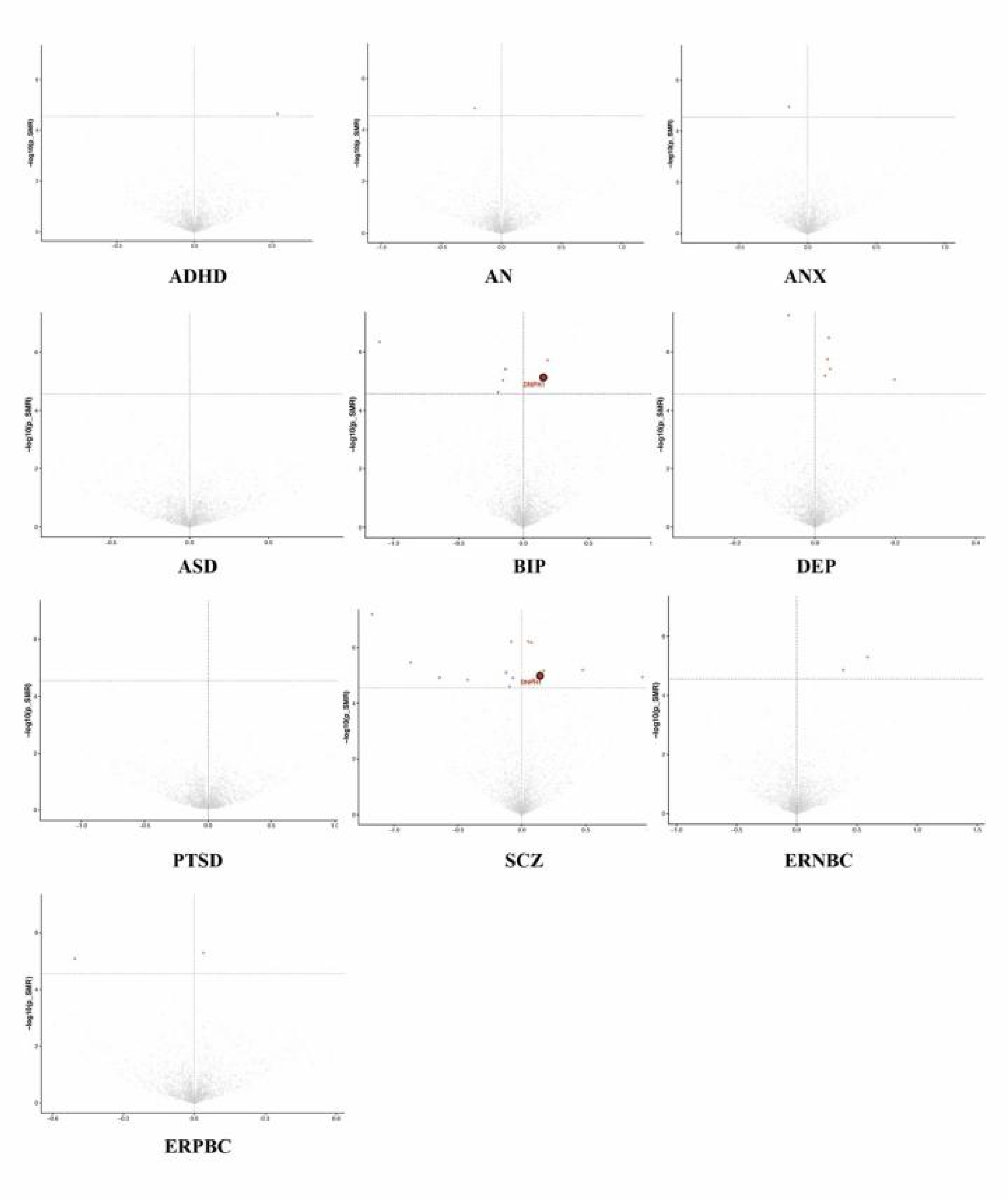

Supplement: Document S1. Figures S1–S3 [file mmc1.pdf]
